# Predictors of Clinical Outcomes of Flexible Ureterorenoscopy with Holmium Laser for Renal Stone Greater than 2 cm

**DOI:** 10.1155/2012/543537

**Published:** 2011-06-09

**Authors:** Saeed M. Al-Qahtani, Sixtina Gil-deiz-de-Medina, Olivier Traxer

**Affiliations:** Department of Urology, Tenon University Hospital, Pierre and Marie Curie University, 4 rue de la Chine, 75020 Paris, France

## Abstract

*Objective*. To evaluate the clinical outcome of flexible ureterorenoscopy (F-URS) with holmium laser in managing renal stone greater than 2 cm. *Patients and Methods*. Records of 120 patients (123 renal units) with renal stone greater than 2 cm who underwent F-URS with holmium laser iwere evaluated. The mean stone size was 26.3 mm. Patient and stone characteristics, perioperative outcomes and complications were evaluated. The outcome was determined at 4 weeks on plain radiograph (KUB) and Non-contrast CT scan (NCCT). Follow-up visit was up to 6 months to evaluate the clinical outcome and patients symptoms. *Results*. Stone burden was an independent predictor of FURS results. After first session of treatment, success rate was obtained in 72 renal units (58.5%). On the other hand, significant residual fragment was encountered in 51 renal units (41.5%). This was improved with “staged-therapy” to 87% and 96.7% after second and third session of treatment, respectively. Complications were recorded. They were managed in proper manner accordingly. *Conclusion*. This is an attractive, safe and effective technique. It is an ideal option for low volume complex stone with average burdens of 2 to 3 cm. Patient should be informed and consented about staged-therapy.

## 1. Introduction

 PCNL has been the standard treatment for renal stone greater than 2 cm [1, 2]. Some authors reported that PCNL stone-free rate is ranging between 77% and 94% [3, 4]. Extracorporeal shock wave lithotripsy (ESWL) for large renal stone renders Stone free (SF) in 51.6% to 57% [5]. Combined ESWL with “RIRS” associated holmium laser were described in comparable results to PCNL [6]. Since Dretler in 1994 [5] described “staged therapy” of F-URS in complex renal stone management, flexible endoscopes underwent great advances in miniaturization as well as durability improvement [5, 7–12]. Many authors reported high success rate between 77% and 92%, minimal morbidity, and short hospital stay of this technique [13–17]. Mariani used electrohydraulic lithotripsy (EHL) in combined with holmium laser reporting promising results with SF rate up to 92% after 2 to 4 sessions of treatment [15]. Holmium laser fiber was used to achieve high stone clearance rate by turning urinary stone into dust, which was eliminated with irrigation fluid [9, 11, 18]. Patients had minimal requirement of pain analgesics and consequently a short hospital stay [19, 20] compared to those patient who were underwent PCNL for almost same burdens of renal stone.

## 2. Patients and Methods

 We reviewed medical records of 120 patients (123 renal units) who underwent flexible ureteroscopy (F-URS) with holmium laser from February 2004 to April 2010. Renal stone size (defined as the largest stone maximal diameter) was ranged from 20 to 58 mm (mean size 26.3 mm). A total of 192 procedures were performed for 120 patients. All patients were operated by a single urologist (O.T) using different types of flexible ureteroscopes: *DUR-8 Elite, Flex-X2, URF-P5 or URF-V. *Holmium laser fibers (150 *μ*m, 200 *μ*m, and/or 365 *μ*) were used according to stone location. Treatment indications were subdivided into certain groups with some overlap among the groups, including ESWL failure, PCNL failure, medical therapy failure, obesity, bleeding disorders as well as some renal congenital malformation (Table 1). Fifty-five renal units (44.7%) were considered as treatment failure of ESWL. Patient age, sex, body mass index, stone size, site, composition, associated lower calyx stone, Double-J stent preoperatively, congenital anomalies, anticoagulant therapy, intervention duration, preoperative serum creatinine and complications were evaluated.

### 2.1. Endoscopic Technique

**Figure 1:**
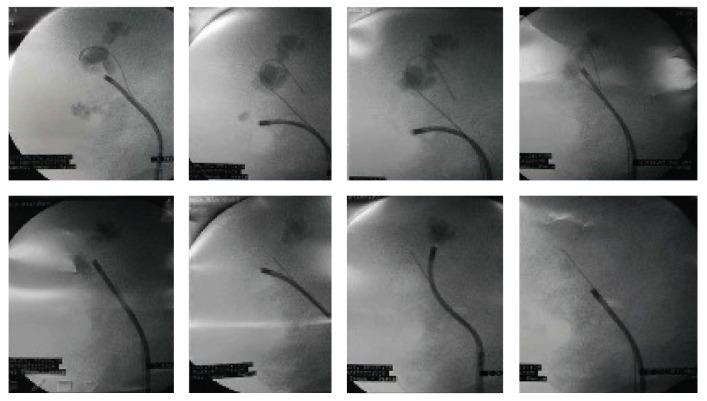
Complete laser fragmentation of multiple renal stones in a single session of F-URS.

**Figure 2:**
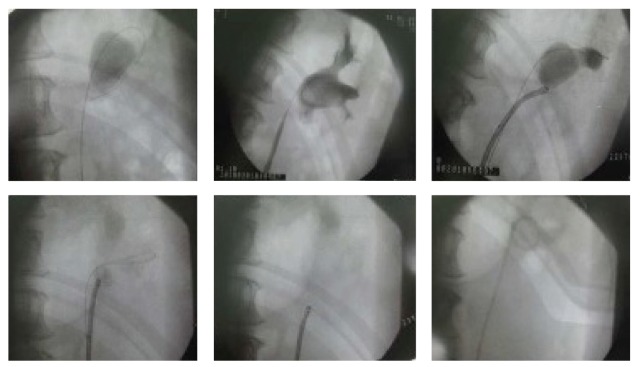
Complete laser fragmentation of large renal pelvis in a single session of F-URS.

 Flexible ureterorenoscopy technique in Tenon University Hospital was performed as described in the literature [8]. In case of complete stone laser fragmentation was obtained as shown in (Figure 1)* (multiple calyceal renal stones) *or in (Figure 2)* (a single renal pelvis stone), *we placed Double-J stent. Zero Tip basket (Boston Scientific) 1.9-Fr was used to extract any visible residual fragments for stone analysis. A ureteral catheter was placed in some patients after a second-look endoscopy, and when we are sure about stone free status, this catheter was removed at Day 1 postoperatively. Patients with uncompleted stone fragmentation were planned for second-look endoscopy and laser fragmentation or for basketing of small fragments within 3 weeks. Second look endoscopies ± multiple stage treatment (starting from 2nd session) were usually performed in day surgery unit in (88%) of patients. In certain situations like solitary kidney and patients who were under anticoagulant therapy, we systemically placed Double-J stent after stone fragmentation. At the end of procedure, a Foley catheter for 24 hr to ensure the maximum drainage. Patients were advised to force fluids to facilitate the physiological evacuation of small fragments. 

 Stone-free status was routinely determined by plain radiograph (KUB) and NCCT at 4 weeks for all patients who had full stone fragmentation. Patients who underwent staged therapy were evaluated at 4 weeks from the last procedure. Success rate was considered as SF or residual fragment of 2 mm or less. All patients were followed up to 6 months, with serial plain radiograph or renal ultrasound (Renal ultrasound at 3 and 6 months). Renal function was compared preoperatively and postoperatively using *t*-test. A metabolic evaluation was performed on patients after stone clearance. All statistical analysis was performed (SPSS, version 16.0).

## 3. Results

 There were 59 male (48%) and 64 female (52.0%). Mean ± SD patient age was 48 ± 15.3 years (median 45, range 19 to 80). Median BMI was 25 kg/m^2^ (range 18.2 to 48.7). (Table 2) shows patients demographic data. SF status was found in 58.5%, 87%, and 96.7% after first, second, and third sessions of treatment respectively. SF status in renal units with stone burden 2 to 3 cm was reported in (65.4%), (90.4%), and (98%) after first, second and third sessions respectively. We performed 192 procedures with mean of 1.6 procedure per patient, including second-look endoscopies. On the other hand, renal units with stone greater than 3 cm rendered SF in (21%) after first session and improved up to (89.5%) by “staged therapy” (up to 3 sessions). 

### 3.1. Outcome Predictors

 On univariate analysis of the entire patient group: stone size category (*P* < .001) and absence of access sheath (*P* = .041) were statistically significantly associated with treatment failure. On multivariate analysis, only stone burden was an independent predictor over the outcome. Large stone burden was related to “staged therapy” (*P* < .0001). Thus, it was associated with residual fragments, and longer operating time. However, no statistically significant association was seen between stone size category and complications (*P* = .8). Renal anatomical abnormalities (*P* = .46) are trended towards a nonsignificant impact over success rate. We reported a horseshoe kidney in 5 renal units (4.3%), a solitary kidney in 9 (7.3%), a caliceal diverticulum in 2 (1.7%) and a pelvic kidney in 2 (1.7%). Lower calyx stone was documented in 71 renal units with no significant impact over results (*P* = .16). There were three patients on anticoagulant therapy; two patients were rendered stone free after first session. Stone analysis was done in all patients (Table 3). In this study, mean operative time was 89 minutes (range 60 to 140). Operative time was significantly correlated to the stone size (*P* < .0001). Nevertheless, it was not affected by stone site (*P* = .35). Hospital stay was ranged between 1 to 3 days (mean = 34.6 hours).

 Preoperative and postoperative renal function (creatinine) was evaluated by *t*-test (*P* = .16), concluding that there was no immediate change in renal function postoperatively which should be also evaluated over the long term.

### 3.2. Complications

 We reported mild-to-moderate Double J stent discomfort in 47% of patients. Those patients were evaluated during hospitalization or at outpatient clinic postoperatively. Complications intra-operatively or postoperatively were reported in Table 4. Three patients had a temporary hematuria postoperatively, which was resolved within 48 hours and none of them was under anticoagulant therapy. Three patients were admitted approximately one week postoperatively (1 pyelonephritis, 1 prostatitis and 1 Obstructive pyelonephritis). All were managed with appropriate antibiotics; for the last patient renal drainage was carried out. We have encountered two patients with steinstrasse phenomenon that treated endoscopically with no major sequences. Subcapsular hematoma was documented in 2 patients who had no preoperative morbidities. Large stone burden was not associated with a greater transfusion requirement neither complication rate.

## 4. Discussion

 PCNL is the standard treatment for large and/or complex stone. However, there are many reports trying to define the minimal stone size for PCNL. Since 1980s, retrograde endoscopic management of upper urinary tract stone had undergone and still undergoing an enormous technical development. In the early 1990s, this technique associated with holmium laser was considered as one of renal stone managment modalities [9, 10]. It is indicated in certain conditions like ESWL treatment failure, medical treatment failure or PCNL failure, obesity, anatomical anomalies as well as bleeding diathesis. ESWL does not carry high success for renal stone greater than 2 cm; moreover, stone-free rate of ESWL for lower pole stone is limited. Several studies were published regarding URS with holmium laser in treating upper urinary tract stone greater than 2 cm or even complex renal stone with comparable results to PCNL [12–17]. Use of actively deflectable ureteroscopes improved exploration of the renal collecting system. Holmium laser lithotripsy introduction into endourological field and stone treatment allowed us to turn urinary stone to small fragments, which are evacuated with the irrigation fluid intraoperatively or in a physiological manner postoperatively [9, 11, 18]. Complication rate of F-URS is not well reported, nevertheless, most of recent studies did not report any major complications [12, 14–17] like perforation or ureteral avulsion compared to semirigid URS [19–21]. PCNL complication rate is reported to be as high as 83% [4, 22], while small size URS had rate of 1.5% [20]. In our series, some complications were recognized. Febrile urinary tract infections were managed appropriately. Postoperatively, the only striking event was the subcapsular hematoma. This was not related to anticoagulant therapy neither to operating time. Over a period of 6-month followup, no ureteral stricture was reported.

 Large stone burden was significantly associated with greater likelihood of repeated sessions, higher probability of residual fragments, and longer operating time. Grasso and associates 1998 [14], obtained 91% stone free with procedure mean of 1.5 for ureteral and renal stones. In regard to operative time, Breda and coworkers [17], reported a mean operative time of 83 minutes (range 45 to 140 minutes) for mean stone burden of 22 mm. Mariani [23] had a mean operative time of 64 minutes (30 to 240 minutes) for mean stone burden of 33 mm using EHL. In this work, the main operative time is 89 minutes (range 60 to 140 minutes). We referred this to two possible factors; first, the presence of lower pole stone in 71 renal units (57.7%) since lower calyx stone related was related long operative time. The second factor was stone nature, since we noticed that those patients with cystine renal stone (15.4%) had longer operative time range from (90 to 124 minutes) with mean of 98 minutes comparing to the other types of stone. The use of F-URS with holmium laser for obese patient is very useful, as many reports described PCNL technically demanding with high risk of complications [19]. We reported 14 renal units (11.4%) in obese patients (BMI > 30), and 78.5% were rendered stone free without any major complications or blood transfusion. 

 Preoperative stenting was not import to address in this study. Some reports suggested that preoperative stents passively dilated the ureter, allowing for high success rate, but there was no report on statistical significant [24]. In this work, the effect of preoperative stenting was not significant in all groups (*P* = .44). For the group 1, 2 cm to 3 cm was also not significant (*P* = .13). The use of access sheath decrease operative time and costs and allow direct insertion of endoscope to the renal cavities with a simple entry and then to visualize renal cavities [25]. It has been shown to extend the durability of ureteroscopes. Nevertheless, complications might occur related to this instrument like ureteral perforation or even stricture over the long term. Delvecchio and coworkers reported the safety of this instrument identifying only one postoperative ureteral stricture from followup of (71) F-URS procedures with an incidence of 1.4% [26].

 The stone size was not homogenously distributed (84.6% of renal units stones are between 2 cm and 3 cm); this was probably due to inclusion criteria (failure of ESWL treatment). We considered this as one of the limits of this study since F-URS is performed as an ancillary procedure after ESWL failure. Due to lack of comparative data, we presented this technique. Up to our knowledge, this is the largest series of patients with stone burdens greater than 2 cm managed by F-URS with holmium laser (Table 5). Our results are comparable, if not superior to, previously published reports of F-URS laser lithotripsy for smaller mean stone burdens and also low rates of recurrence, although most series have limited followup like this study. From this study, we emphasize that F-URS with holmium laser lithotripsy is safe and effective for management of 2 to 3 cm renal stones. Nevertheless, Retreatment rate for this group (2 to 3 cm) was 34.6%, which is less than other reports. Success rate was strongly associated with stone size; therefore, “staged therapy” should be carefully discussed with the patient who has a large-volume renal stone. Moreover, success rate for renal stone (greater than 3 cm) reached almost 90% after 3 sessions.

## 5. Conclusions

 As PCNL remains the golden standard for large renal stone, F-URS with holmium laser is an attractive safe and effective technique with low morbidity and high success rate. Therefore, it could be proposed as a real alternative therapy to PCNL for certain indications (group of high risk). However, this technique is an ideal option for low-volume complex stone with average burdens of 2 to 3 cm. Patient should be informed and consented about staged-therapy. Further comparative studies should be conducted between groups of patients with certain indications to lineate the best treatment option.

## Figures and Tables

**Table 1:** Treatment indications associated with treatment success.

Treatment indication	No. RU	Success no. of RU
ESWL treatment failure	54	51 (98%)
PCNL treatment failure	14	12 (85.7%)
Medical treatment failure	14	11 (78.5%)
Medical comorbidities	16	9 (75%)
Bleeding disorders	3	3 (100%)
Morbid obesity	14	11 (78.5%)
Solitary kidney	9	9 (90%)
Pelvic kidney	2	2 (100%)

RU: Renal Unit.

**Table 2:** Patients' demographics data and association to treatment success.

Variable	Results	*P*	Test
Age (Mean ± SD) years	48.0 ± 15.3	.63	Mann-Whitney
Gender		.71	Fisher's exact
Female	64 (52.0%)		
Male	59 (48.0%)		
BMI (Mean ± SD)	25.4 ± 6.1	.29	Mann-Whitney
Malformation (RU)		.46	Chi-square
Horseshoe kidney	5 (4.3%)		
Solitary kidney	9 (7.3%)		
Pelvic kidney	2 (1.7%)		
Caliceal diverticula	2 (1.7%)		
Malrotated kidney	1 (0.9%)		
Repaired UPJ obstruction	4 (3.4%)		
None	100 (8.3%)		
Anticoagulant therapy		.91	Fisher's exact
Yes	3 (2.4%)		
No	120 (97.6%)		

RU: renal unit, SD: standard deviation.

**Table 3:** Stone Demographics characteristics, preoperative and intraoperative data.

Variable	Results	*P* value	Test
Size (SD) mm	26.3 ± 7.3	.001	Fisher's exact
20–30	104 (84.6%)		
>30	19 (15.4%)		
Associated lower calyx stone		.16	Fisher's exact
Yes	71 (57.7%)		
No	52 (42.3%)		
Site (RU)		.85	Chi-square
Pelvic	22 (17.9%)		
Lower calyx	13 (10.6%)		
Middle calyx	1 (0.8%)		
Upper calyx	2 (1.6%)		
Mixed pelvic and calyces	67 (54.5%)		
Mixed calyces	18 (14.6%)		
Stone Nature		.22	Chi-square
Ca oxalate	71 (57.7%)		
Mixed	9 (7.3%)		
Uric acid	7 (5.7%)		
Brushite	11 (9.0%)		
Struvite	6 (4.9%)		
Cystine	19 (15.4%)		
Stone number		.81	Fisher's exact
Isolated	53 (43.1%)		
Multiple	70 (56.9%)		
Preoperative DJ stent		.44	Fisher's exact
Yes	81 (65.9%)		
No	42 (34.1%)		
Operative time (minute)	89 ± 24	.34	Mann-Whitney
Use of ureteral access sheath	117 (95.1%)	.041	Fisher's exact

**Table 4:** Intraoperative and postoperative complications.

Intraoperative	
Bleeding	1
Perforation (renal pelvis)	1
Fornix rupture	3

Postoperative	

Prostatitis	1
Non obstructive pyelonephritis	1
Obstructive pyelonephritis	1
Steinstrasse	2
Subcapsular hematoma	2
Temporary hematuria	3

**Table 5:** Literature review for flexible ureteroscopic and holmium laser management for stone larger than 2 cm.

Study	No. Pts	Mean stone size mm	Average number of procedure	SF%
Riley et al. [27]	22	24	1.82	90.9
Breda et al. [17]	15	22	2.3	93.3
Grasso et al. [14]	51	26.7 (renal)	1.3	91
El-Anany et al. [16]	30	NM	1	77
Our series	120	26	1.6	95.9

NM: not mentioned, SF: stone free.

## Abbreviations

PCNL: Percutanous nephrolithotomy
F-URS: Flexible ureterorenoscopy
SF: Stone free
BMI: Body mass index
CT: Computerized tomography
RIRS: Retrograde intra-renal surgery
ESWL: Extracorporeal shock wave lithotripsy
